# Increased Incidence of Methicillin-Resistant Staphylococcus aureus Skin and Soft Tissue Abscesses in Indigenous Children in North Queensland

**DOI:** 10.7759/cureus.105132

**Published:** 2026-03-12

**Authors:** Ezekiel Aaron, David Kanaganayagam, Helen Buschel, James Carroll, John Avramovic, Brendan R O'Connor

**Affiliations:** 1 General Surgery, Townsville University Hospital, Townsville, AUS; 2 Paediatric Surgery, Townsville University Hospital, Townsville, AUS; 3 Medicine, James Cook University, Townsville, AUS; 4 General Surgery, North Queensland Minimally Invasive Surgery, Townsville, AUS

**Keywords:** aboriginal and torres strait islander, empirical antibiotic therapy, mrsa abscess, paediatric surgery, regional australia, surgical incision and drainage

## Abstract

Introduction

The rise of Methicillin-resistant *Staphylococcus aureus* (MRSA) is a major concern in Australia, particularly affecting Aboriginal and Torres Strait Islander (Indigenous) people. This study aimed to identify the incidence of MRSA in Indigenous compared to non-Indigenous children with skin and soft tissue abscesses (SSTAs).

Methodology

Following ethical approval, electronic hospital records were retrospectively reviewed to identify microbiological swabs from patients who underwent incision and drainage of SSTAs at Townsville University Hospital between January 2020 and December 2024. Patients under 16 years with their ethnicity data were included.

Results

Four hundred and fifty-two children met the inclusion criteria. Two hundred and thirty-four (52%) identified as Indigenous and 218 (48%) as non-Indigenous. MRSA was isolated in significantly more Indigenous (41%, 96/234) than non-Indigenous (21%, 45/218) patients (p<0.001). Indigenous children were 2.7 times more likely to have MRSA (OR: 2.7, 95% CI: 1.9, 3.8). Methicillin-sensitive *Staphylococcus aureus *(MSSA) was cultured in 38% (174/452) of samples. Other cultured organisms included *Streptococcus* species (10%), mixed skin flora (5%), and mixed enteric and anaerobic bacteria (4%). Other multi-resistant organisms included Extended-Spectrum Beta-Lactamase-producing *Escherichia coli (E. Coli)* (1/452) and *Mycobacterium abscessus* (1/452). SSTAs most commonly affected the limbs (29%), followed by the head and neck (23%), buttocks (15%), torso (9%), perianal/perineal (9%), and groin (6%). Most SSTAs were drained by the paediatric surgeons (70%), followed by orthopaedic surgeons (18%), with smaller numbers by maxillofacial, ear-nose and throat, and general surgeons.

Conclusion

We identified higher rates of MRSA in Indigenous children with SSTAs compared to non-Indigenous children, justifying empiric MRSA antimicrobial coverage for Indigenous children. Future prospective studies should include follow-up to assess rates of recurrence, side effects of antibiotics, and whether the same trend continues into adulthood.

## Introduction

*Staphylococcus aureus *is a common commensal skin flora. But it can breach the epithelial barrier to develop deeper skin and soft tissue infections. Infections range from cellulitis to skin and soft tissue abscesses (SSTAs). SSTAs can disseminate into the bloodstream, leading to sepsis and infections of other organs [1].

Methicillin-resistant *Staphylococcus aureus *(MRSA) refers to strains resistant to penicillins and other beta-lactams [2]. MRSA has a mecA gene, which encodes an altered penicillin-binding protein that reduces its affinity to beta-lactam antibiotics that are traditionally used to treat skin infections [3]. It was initially noticed in hospital patients, but new community-acquired strains of MRSA (CA-MRSA) have led to an epidemic in Australia, especially in Indigenous communities [4].

The new variants have shown hypervirulence leading to more severe infections via abscess formation, septicaemia, and necrotising pneumonia [5]. This has been attributed to a virulence factor called Panton-Valentine leucocidin (PVL). PVL is an endotoxin that mediates leukocyte recruitment and tissue destruction. Consequently, carriage of MRSA not only increases the risk of developing SSTAs but also increases the risk of the associated complications [5]. 

Studies done on school-aged children in Indigenous communities in Queensland (QLD) have reported MRSA carriage rates as high as 12-15%. A large proportion had the PVL virulence factor [6, 7]. A New South Wales (NSW) study looking at *Staphylococcus aureus *clinical specimens collected at two major hospitals demonstrated a 2.6 times higher rate of MRSA in Indigenous patients compared to non-Indigenous patients [8]. Indigenous Australians have the highest rates of hospitalisation due to skin infections. In some regions, they had a 10-fold increase in rates of sepsis and osteomyelitis from staphylococcal infections compared to non-Indigenous Australians [9].

MRSA prevalence has been difficult to predict with variation over time and geography. Temporospatial mapping of AusLab microbiology data from 1997 to 2016 demonstrated large changes in MRSA rates in *Staphylococcus aureus* isolates across Far North Queensland. Rates ranged from up to 73% in the Cook Town region to less than 5% in the Cape York Peninsula. This is due to variations in the local microbiome as well as differences in socioeconomic and Indigenous population size in different regions. The AusLab data showed that over time, the rates of MRSA increased in North Queensland [10]. However, their data did not capture the Townsville region and only included clinical isolates collected up until 2016. This study looked at the Townsville region and explored MRSA rates from 2020 to 2024. The study aimed to capture any patterns or changes in MRSA rates over a more recent time period. 

Many Australian statewide paediatric guidelines recommend empirically treating Indigenous children who have SSTAs with antimicrobials that have MRSA coverage. The two main antibiotics recommended are sulfamethoxazole/trimethoprim (Bactrim) and clindamycin [11, 12]. This practice is also empirically followed at Townsville University Hospital, in the absence of local data to confirm the generalisability of the AUSLAB data to Townsville, which we aimed to address in this study. The endemic nature of MRSA carriage in Indigenous communities has been established by small case-control studies in Queensland. However, there have not been any larger studies in more urban areas investigating specifically how this increase in MRSA carriage translates to hospital presentations of SSTAs in Queensland. This study will provide guidance on antimicrobial prescribing for paediatric patients with SSTAs and determine whether Indigenous patients should have different antibiotic recommendations.

## Materials and methods

Study population

This is a single-centre retrospective cohort study. The study was conducted at Townsville University Hospital. Townsville is the largest city in North Queensland and has a population of 234,282 people [13]. Aboriginal and/or Torres Strait Islander people make up 9% of the total population. Sixteen percent of the children under the age of 14 are of Indigenous heritage [13].

Townsville University Hospital is one of the largest tertiary hospitals in North Queensland. Its catchment area stretches over 148,000 square kilometres, containing around 700,000 people. It stretches from Cardwell to the North, Home Hill to the South, Palm Island to the East and Hughenden to the west [14].

Children under the age of 16 who required an incision and drainage for community-acquired skin or soft tissue abscess in the operating theatre from January 2020 to December 2024 were included in this study. Patients who developed SSTAs during an inpatient admission (i.e., hospital-acquired SSTAs) were excluded.

Data collection

Data collectors used the hospital’s electronic medical records (iEMR) to systematically collect data for eligible patients, including demographic data, ethnicity, site of drainage, specialist surgical team involved, antibiotics prescribed, and pre- or intraoperative swab cultures, including the organism and sensitivities.

Every patient admitted to Townsville University Hospital is routinely asked whether they are an Aboriginal and/or Torres Strait Islander by administration staff. This information is recorded and can be viewed on iEMR in the patient's files. 

A skin and soft tissue abscess was defined as a collection of purulent material described in the intraoperative findings or preoperative assessment. Infections spreading deeper to muscle, tendon, or bone were not included. If multiple abscesses were drained during the same procedure, the largest abscess determined from the operation notes was included. Abscesses that developed in postoperative wounds were not included in this study. Patients who had repeat admissions for SSTAs were analysed as independent cases for each separate encounter. 

Some patients, despite having clinical SSTAs, did not have swabs collected. This was dependent on surgeon preference. Given the retrospective nature of the study, this factor could not be controlled. These patients were still included in the statistical analysis of the results. 

Microbiology

Swabs were sent to the Townsville Hospital Pathology lab for standard laboratory testing. Bacterial isolates and antibiotic sensitivity testing were performed. This microbiological data was recorded in the Queensland statewide pathology laboratory information system (AusLab).

Data from AusLab, including organisms cultured and sensitivities, were then tabulated. Polymicrobial growths, such as mixed skin flora, were counted as 1 single case in organism frequency calculations. Specific genotyping and PCR testing were not performed on bacterial isolates, including MRSA, at the Townsville Hospital Laboratory. 

Statistical analysis

Statistical analysis was performed on the tabulated unadjusted data using Excel software (Microsoft, Redmond, Washington). Basic percentage data was calculated. The Chi^2^ test was used to measure statistical significance. A p-value less than 0.05 was considered statistically significant. In order to calculate a Chi^2^ value, a null hypothesis was formulated. The null hypothesis stated that Indigenous and non-Indigenous patients would have an equal distribution of MRSA. An odds ratio and corresponding confidence intervals were also calculated using Excel software. Age, co-morbidities, and other confounding factors were not adjusted for during the statistical analysis. 

Ethics

The ethics proposal was approved by the hospital's Audit, Quality and Innovation Review Panel (AQUIRE). The project was formulated with consultation from the Paediatric Surgical and General Surgical Teams at the Townsville University Hospital. The data collection and analysis were performed by a Surgical Principal House Officer and Registrar working as part of the Paediatric Surgical Team. Data collection was performed over a one-year period in 2025. Data was securely stored in a password-locked OneDrive spreadsheet (Microsoft, Redmond, Washington) and was only accessible to the project team. All data published was de-identified, maintaining confidentiality for patients. Data will be stored for 10 years after the study completion on a Research Data Lifecycle (RDL) Data Archival Service. 

## Results

Participant characteristics

**Figure 1:**
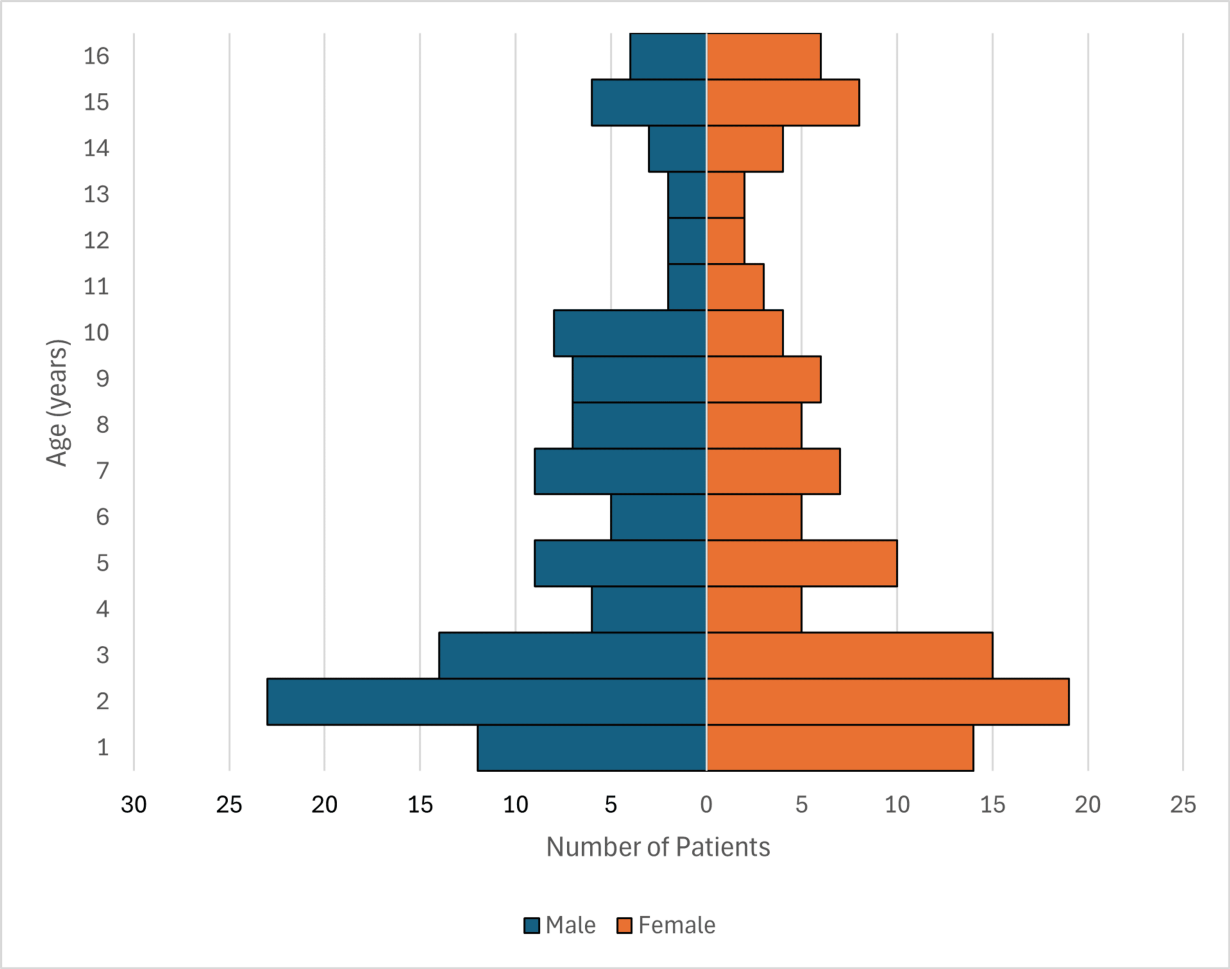
Age and sex distribution in Indigenous patients

**Figure 2:**
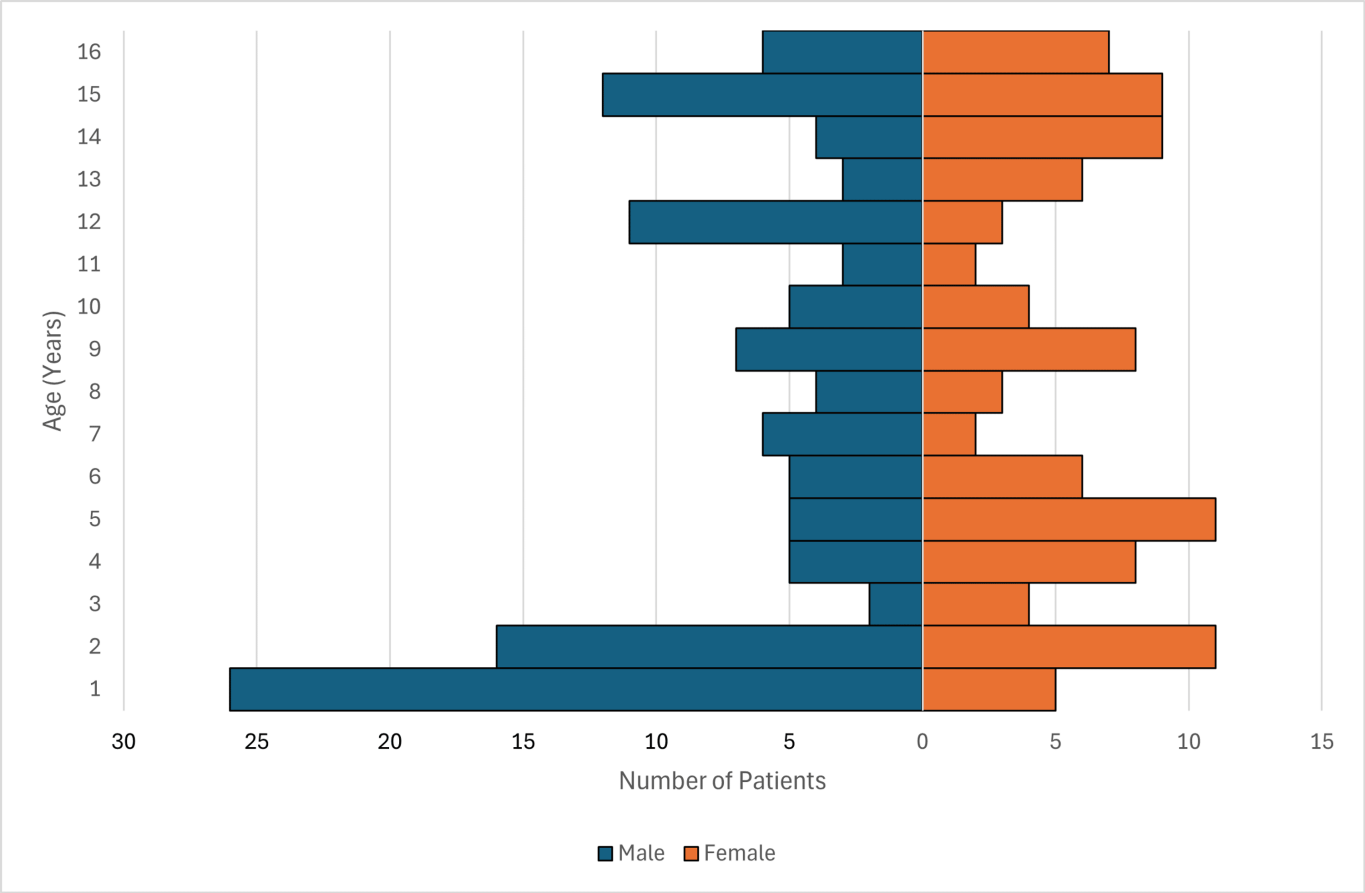
Age and sex distribution in non-Indigenous patients

There were 452 children who met the eligibility criteria and were included in this study. All children were under the age of 16. SSTAs were more prevalent in younger children, with the largest age category being children less than two years of age (126 patients, 28% of the total study participants). The mean age was six years (SD=5; range: 0-15). Forty-seven percent(213) of patients were female, and 53% (239) were male. Fifty-two percent (234) of patients were Indigenous. This included people who identified as Aboriginal (147, 33%), Torres Strait Islander (22, 5%), and both (64, 14%). There was only one South Sea Islander. The other 48% (218) of patients were non-Indigenous. The age and sex distribution for Indigenous and non-Indigenous patients are shown in Figure 1 and Figure 2, respectively. 

Sites of SSTAs

**Figure 3:**
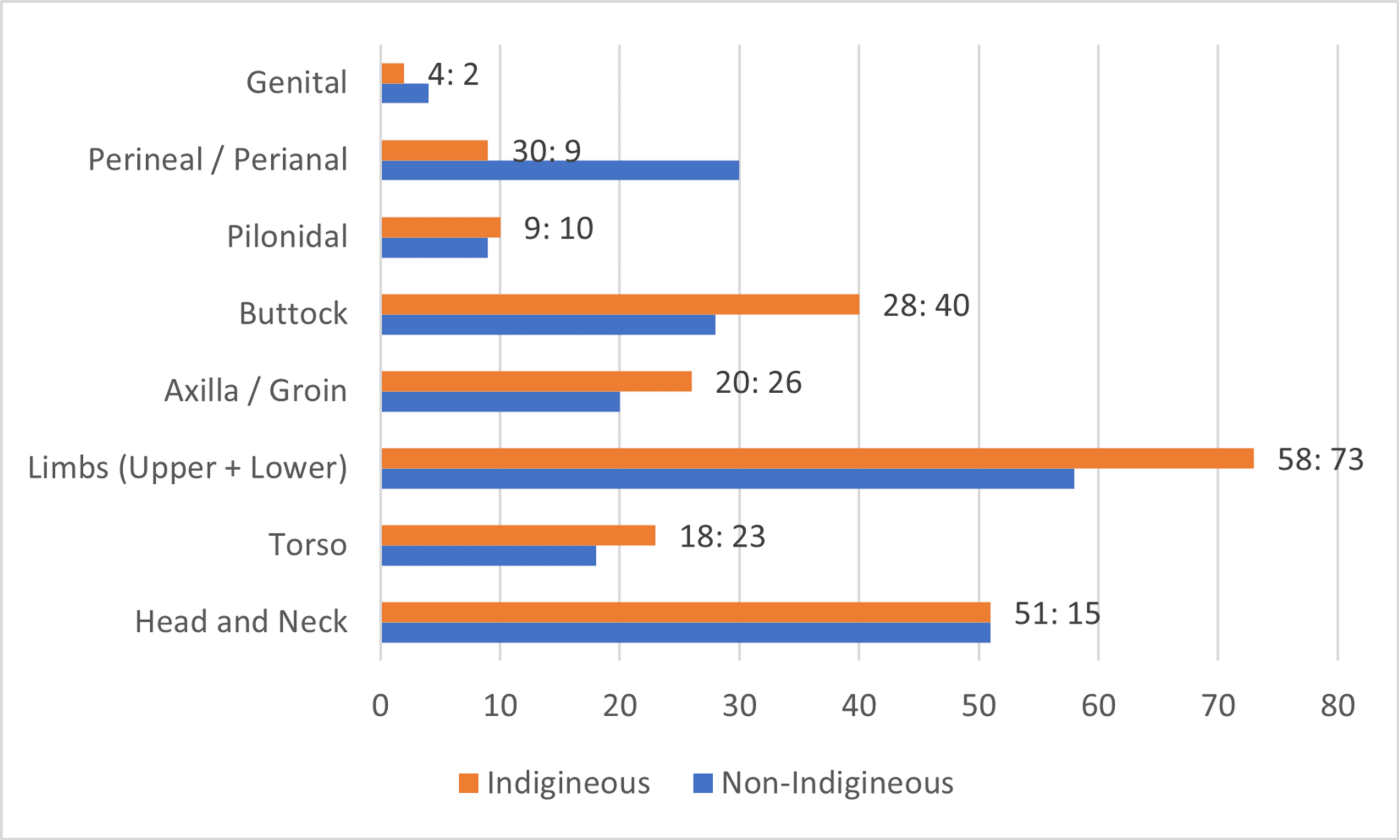
SSTA distribution in Indigenous and non-Indigenous patients Genital SSTAs X^2^ (1, n=6) = 0.67, p=0.41; Perineal / Perianal SSTAs X^2^ (1, n=39) = 14.45, p<0.001; Pilonidal SSTAs X^2^ (1, n=19) = 0.05, p=0.82; Buttock SSTAs X^2 ^(1, n=68) = 2.12, p=0.15; Axilla / Groin SSTAs X^2^ (1, n=46) = 0.78, p=0.38; Limb SSTAs X^2^ (1, n=131) = 1.72, p=0.19; Torso SSTAs X^2 ^(1, n=41) = 0.61, p=0.43; Head and Neck SSTAs X^2^ (1, n=102) = 0, p=1.00

Abscesses were widely distributed throughout the body. The most common sites being upper and lower limbs (131 cases, 29%), followed by head and neck (100 cases, 23%), buttocks (68 cases, 15%), intertriginous areas i.e., axilla and groin (46 cases, 10%), torso (41 cases, 9%) and then the perineal and perianal regions (39 cases, 9%). Both groups had 51 cases of head and neck abscesses. Non-Indigenous patients had more genital, perineal, and perianal abscesses with statistically significant differences in the perineal and perianal abscesses combined. There were 30 cases of perineal and perianal abscesses in non-Indigenous patients and nine cases amongst the Indigenous patients (X^2 ^(1, N=39) = 10.94, p<0.001, OR: 4.9, CI: 2.1, 9.0). Indigenous patients had more pilonidal, buttock, axilla, groin, limb, and torso abscesses, but there were no statistically significant differences shown (p>0.05) as seen in Figure 3. 

Recurrent presentations

Twelve patients within the five-year period of this study had multiple incisions and drainages of abscesses in subsequent hospital admissions. One patient had three different admissions for incision and drainage of perianal abscesses. Nine of the 12 patients cultured *Staphylococcus aureus*, five of whom cultured MRSA. Nine of the patients were non-Indigenous, and three were Indigenous. Each new admission was counted separately during the statistical analysis. 

Bacterial organisms

There were 22 different bacterial species cultured, not including the polymicrobial growths such as mixed anaerobic bacteria, mixed enteric bacteria, mixed oral flora, and mixed skin flora. The most common organism cultured was *Staphylococcus aureus,* which was present on 300 cultures (66%). This included MRSA, which accounted for 141 cultures (31%). The majority of *Staphylococcus aureus* was methicillin-sensitive but resistant to penicillin G (159, 35%). There were 28 patients who either had no growth from their cultures (23) or no swabs taken (five). All 28 patients were included in the organism frequency calculations. There were 15 cases of pan-sensitive *Staphylococcus aureus*. Eleven of these 15 patients were non-indigenous (X^2^ (1, N=15) = 3.27, p=0.07). Other common bacterial species included *Streptococcus s*pecies (spp) (45 cases, 10% of SSTAs), *Pseudomonas spp*, *Bacteroides spp,* and *E. Coli *(six cases each), as seen in Table 1. 

**Table 1:** Cultured organisms Most organisms, except for* Staphylococcus aureus and Escherichia coli,* were grouped into species for this table. PSSA - pan-sensitive Staphylococcus aureus; MRSA - methicillin-resistant *Staphylococcus aureus*; MSSA - methicillin-sensitive *Staphylococcus aureus*

Swab results	Total number of patients
Actinomyces sp	1
Capnocytophaga sp	1
Citrobacter sp	1
Moraxella sp	1
Mycoplasma sp	1
Pasteurella sp	1
Peptoniphilus sp	1
Prevotella sp	1
Haemophilus spp	2
Klebsiella spp	2
Propionibacterium spp	2
Trichophyton spp	2
Mycobacterium spp	3
No swab	5
Bacteroides spp	6
Escherichia coli	6
Pseudomonas spp	6
Mixed oral flora	7
Mixed anaerobic bacteria	9
Mixed enteric bacteria	11
PSSA	15
No growth	23
Normal skin flora	24
Streptooccus spp	45
MRSA	141
MSSA	159

MSRA prevalence

**Figure 4:**
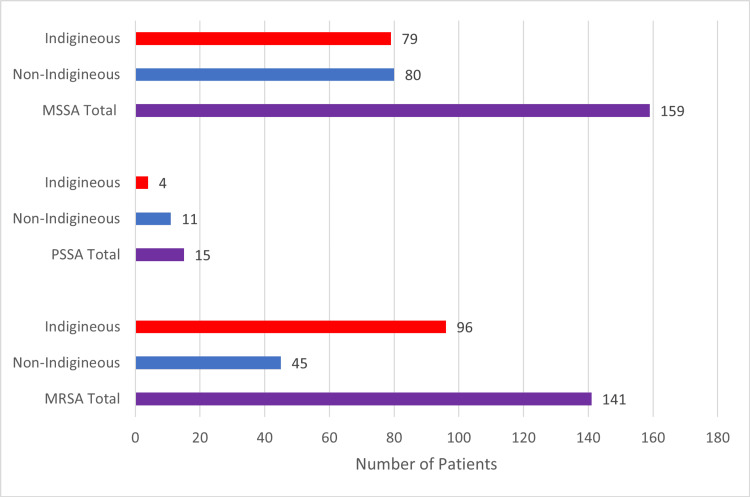
Staphylococcus aureus breakdown in Indigenous and non-Indigenous patients MSSA - methicillin-sensitive Staphylococcus aureus; PSSA - pan-sensitive Staphylococcus aureus; MRSA - methicillin-resistant Staphylococcus aureus

A higher proportion of patients with MRSA were indigenous (96 patients, 68% of MRSA cases) compared to non-Indigenous (45 patients, 32%), as seen in Figure 4. Overall, 41% (96/234) of Indigenous patients presenting with SSTAs had MRSA compared to 21% (45/218) of non-Indigenous patients. (OR: 2.7, CI: 1.9, 3.8) This difference was statistically significant (X^2 ^(1, N=141) = 15.02, p<0.001). Besides MRSA, other multi-resistant organisms included one case of extended-spectrum beta-lactamase-producing *Escherichia coli* and one case of *Mycobacterium abscessus*.

**Figure 5:**
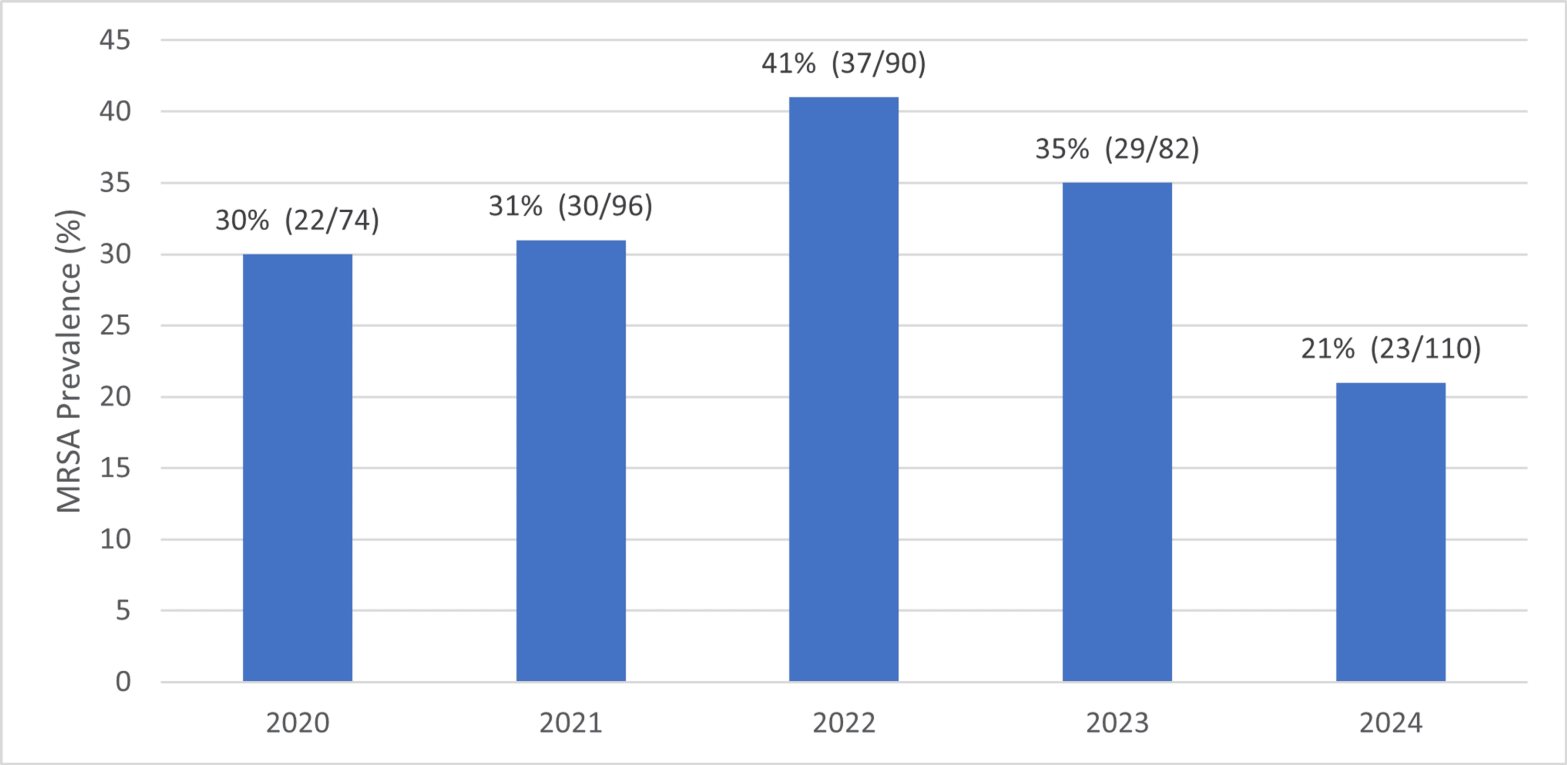
MRSA prevalence as a percentage of the annual total number of SSTAs over the five-year study period MRSA - methicillin-resistant Staphylococcus aureus; SSTAs - skin and soft tissue abscesses

Over the five-year study period, rates of MRSA slowly increased to peak in 2022 at 41% (37/90) of SSTAs requiring an incision and drainage at Townsville University Hospital. This reduced over time to the final year of the study period to its lowest rate of 21% (23/110) in 2024, as seen in Figure 5. 

Surgical specialities involved

Abscesses were drained by multiple specialties. Most abscesses were drained by the paediatric surgical team, who did 315 (70%) of the 452 cases. Other specialties included orthopaedic, ear-nose and throat, and maxillofacial surgery.

## Discussion

Several studies have looked at MRSA skin carriage and prevalence in skin lesions and skin infections. There is a scarcity of data from QLD specifically looking at MRSA infections causing SSTAs. Studies in North Queensland have mainly been community-based with small cohort sizes [6, 7]. This study had a larger cohort size and was uniquely based in a major regional city.

In the 2021 Census, Aboriginal and/or Torres Strait Islander people made up 9% of the Townsville population [13]. Indigenous people in major cities generally have a higher socioeconomic status than those living in more rural and remote parts of Australia [15]. The differing population density and socioeconomic status of Indigenous people living in Townsville could lead to different rates of MRSA compared to other parts of Queensland. For these reasons, despite having previously established statewide antibiotic guidelines, it is still important to tailor local guidelines to the current MRSA prevalence in the community.

Indigenous children were overrepresented in this study cohort compared to their distribution in the general population. Using the 2021 Census data, 16.3% of children living in Townsville under the age of 15 were Indigenous. This is significantly less than this study cohort, where 52% (224/429) of children under the age of 15 presenting with SSTAs were Indigenous (X^2 ^(1, N=429) = 404.86, p<0.001) [13]. Indigenous children had an 8.6 times greater relative risk of SSTAs that required incision and drainage compared to non-Indigenous children in 2021 (OR: 8.67, 95% CI: 6.08 - 12.35) [9, 16]. 

Indigenous children in the study had a 2.7 times higher rate of MRSA in swabs collected from SSTAs. This pattern was also reflected in other studies performed in Australia. Agostino et al demonstrated a 2.6 times higher rate of MRSA in Indigenous patients from *Staphylococcus aureus* isolates collected from clinical specimens in NSW [8]. In North Queensland, smaller community studies reported a 13-15% rate of MRSA carriage in Indigenous children from skin and nasal swabs [6, 7]. In contrast, this study recorded 41% of Indigenous patients culturing MRSA. The higher rates of MRSA were because specimens were collected directly from abscesses, whereas the community-based studies only looked at nasal and skin carriage. A similar study in Alice Springs in central Australia found an even higher rate of MRSA of 57% in a cohort of mainly Indigenous patients presenting with skin and soft tissue infections [16].

There are many social determinants of health underpinning health disparities between Indigenous and non-Indigenous Australians. Pertaining to MRSA, overcrowding, poor hygiene and sanitation, lack of access to laundry facilities and running water, propagate the spread and persistence of MRSA in these communities [17, 18]. Indigenous Australians also have higher rates of hospitalization and exposure to antibiotics. This leads to antimicrobial pressures that promote resistant organisms such as MRSA [19]. Indigenous children have higher rates of skin lesions such as scabies, impetigo, and other pruritic skin conditions, such as eczema [9]. These conditions compromise the protective skin barrier, predisposing them to skin and soft tissue infections. Additionally, a study done in Townsville, Queensland, indicated that Indigenous children may have a weakened immune system due to a variety of factors, including malnutrition and obesity. These health factors could make them more susceptible to SSTAs with higher risks of complications [20].

This research supports the need for greater effort at preventative strategies at the community level to address this health disparity. Some interventions that have shown success include community swimming pools and education and early treatment of skin lesions in the Indigenous population [17, 21]. With the use of the already established Aboriginal and/or Torres Strait Islander Health Services, the implementation of such strategies at a primary health care level is possible.

The findings support MRSA antimicrobial coverage for Indigenous children with SSTAs. Interestingly, the rate of MRSA in non-Indigenous study participants was also high at 21% (45/218). This raises the question of whether non-Indigenous children with SSTAs should also have empirical MRSA cover. However, increasing the use of non-beta-lactam antibiotics can promote antimicrobial resistance and the development of multi-resistant MRSA.

Most strains of MRSA were resistant to penicillin G, flucloxacillin, and cefalexin. Thirteen study participants had additional resistance to clindamycin (two) and Bactrim (11). This made up 4% (13/315) of the total* Staphylococcus aureus* isolates. This resistance profile is similar to Queensland AUSLAB data records in 2016, where 2% of *Staphylococcus aureus* isolates were resistant to Bactrim, and 9% were resistant to clindamycin [10]. All 13 of these patients were Indigenous (X^2 ^(1, N=13) = 13, p<0.001). On the other hand, there were 15 cases of pan-sensitive Staphylococcus aureus (PSSA). Eleven of these 15 patients were non-Indigenous (X^2 ^(1, N=15) = 3.27, p=0.07).

The general trend towards more resistant *Staphylococcus aureus *strains in Indigenous children could be due to higher antimicrobial pressures from increased skin and upper respiratory tract infections compared to non-Indigenous children [22]. In Australia, up to 45% of Aboriginal children living in remote regions of Australia have impetigo [9]. The significance of early life exposure to antibiotics has been demonstrated in in-vitro studies, which have been able to induce resistance to Bactrim in *Staphylococcus aureus *cultures as early as day eight of antibiotic exposure [23]. The developing resistance to even non-beta-lactam antibiotics raises the question of whether simply prescribing the correct antibiotic should be the only hospital's long-term strategy for reducing MRSA rates.

Abscesses involving the oral cavity, such as buccal, sub-maxillary, and submandibular abscesses and deep neck abscesses involving parapharyngeal and retropharyngeal structures were also included in this study. MRSA is usually not the culprit of such infections. There were 11 cases of intraoral or deep neck abscesses. None of these cases had cultured *Staphylococcus aureus*. The most common pathogens were normal/mixed oral flora, indicating a different route of infection. It could be argued that in future studies evaluating SSTAs, intraoral and deep neck abscesses should be excluded. 

A limitation of this study includes possible selection bias, as only patients requiring incision and drainage of SSTAs in the operating theatre were included. This excludes a potentially large number of patients who had drainage procedures outside of theatre, including in the emergency department and in the primary health care setting. Furthermore, confounding factors such as age, sex, and comorbidities were not adjusted for. Statistical analysis was performed on crude/unadjusted data and hence did not account for the effects of such confounding factors, which could introduce bias into the results. 

Twelve patients had recurrent admissions for SSTAs. Each new admission was counted separately during the data analysis. Consequently, this data was not independent and did not come from a unique study participant, potentially introducing bias. In future research, statistical methods should be used that account for clustered data. Furthermore, patients with no swabs collected were also included in the statistical analysis. However, this was a small number of patients (five) and hence should not greatly impact the overall calculated rates of MRSA. In future prospective research, a standardized approach to collecting tissue cultures and swabs should be used by participating surgeons. 

Any complications from SSTAs, such as sepsis, osteomyelitis, and pneumonia, were not recorded. Furthermore, data on patients requiring repeat surgical drainage during the same admission or failed treatment were not recorded. This information should be collected in future research to evaluate the efficacy of antibiotic use. However, patient outcomes also depend on various other factors, including comorbidities and the efficacy of the drainage procedure. Hence, a standardized management approach needs to be devised to accurately assess the outcomes of treatment. In future research, genotyping and PCR testing should be performed to identify specific strains of MRSA and virulence factors such as PVL. This would provide a better understanding of the epidemiology and pathophysiology of local MRSA strains. Now that this study has supported MRSA antibiotic coverage in the Indigenous population, antibiotic use should be audited, especially given the risk of developing antimicrobial resistance.

Pertinent risk factors such as personal or family history of previous skin infections, history of MRSA, pre-hospital antibiotics, overcrowding, impetigo or scabies, and other comorbidities were not recorded. This was difficult to obtain as there was no standardized questionnaire used for history, since this study was performed retrospectively. In future prospective studies, including such data would be useful in determining the impacts of certain social, environmental, and biological risk factors on SSTAs to provide further guidance on antibiotic prescribing. This would also inform primary prevention strategies to reduce the upstream factors contributing to SSTAs.

This study only looked at SSTAs in children. Hence, it cannot be generalised to the adult population. MRSA rates should be explored in the adult population to see if this trend continues in older age groups. 

Despite some of this study's methodological limitations, the results show significant trends that support antimicrobial coverage for MRSA in Indigenous children. This study on its own has relatively weak statistical strength and power, but when taken in the context of the overwhelming evidence demonstrated nationwide, it can be used to support already established guidelines. Furthermore, the study's findings aim to stimulate further prospective research with more robust methodology and statistical analysis to provide stronger evidence behind MRSA antimicrobial coverage in Indigenous patients. 

## Conclusions

We identified higher rates of MRSA in Indigenous children with SSTAs compared to non-Indigenous children in our population. In this context, antibiotics with MRSA coverage should be used for empirical treatment of Indigenous children. The study also identified higher rates of MRSA in non-Indigenous children. Further research should be done to evaluate whether non-Indigenous children with SSTAs should also have MRSA coverage. However, this could lead to the development of increasingly more resistant strains of MRSA. In fact, several of the MRSA cultures were resistant to Bactrim and clindamycin. This suggests appropriate antibiotic prescribing may not be the only solution to prevent progression of antimicrobial resistance. Further research should be done looking at biological and social risk factors underpinning the higher prevalence of MRSA to inform primary health and community strategies to reduce this health disparity among Indigenous Australians. Further, prospective studies should be performed following up patients to see their outcomes, including rates of SSTA recurrence and side effects from antibiotic use. Similar studies can be extended to include the adult population to see if the same trend continues. 
